# Transmission of the *Aegilops ovata* chromosomes carrying gametocidal factors in hexaploid triticale (×*Triticosecale* Wittm.) hybrids

**DOI:** 10.1007/s13353-015-0332-3

**Published:** 2016-01-29

**Authors:** M. Kwiatek, M. Majka, A. Ślusarkiewicz-Jarzina, A. Ponitka, H. Pudelska, J. Belter, H. Wiśniewska

**Affiliations:** Institute of Plant Genetics, Polish Academy of Sciences, Strzeszyńska 34, 60-479 Poznań, Poland

**Keywords:** *Aegilops*, Chromosome, Gametocidal factor, Meiosis, Preferential transmission, Triticale

## Abstract

The main aim of this work was to induce the chromosome rearrangements between *Aegilops ovata* (UUMM) and hexaploid triticale (AABBRR) by expression of the gametocidal factor located on the chromosome 4M. The *Aegilops ovata* × *Secale cereale* (UUMMRR) amphiploids and triticale ‘Moreno’ were used to produce hybrids by reciprocal crosses. Chromosome dynamics was observed in subsequent generations of hybrids during mitotic metaphase of root meristems and first metaphase of meiosis of pollen mother cells. Chromosomes were identified by genomic in situ hybridisation (GISH) and fluorescence in situ hybridisation (FISH) using pTa71, pTa791, pSc119.2 and pAs1 DNA probes. It has been shown that the origin of the genetic background had an influence on *Aegilops* chromosome transmission. Moreover, it has been reported that the preferential transmission of chromosome 4M appeared during both androgenesis and gynogenesis. It is also hypothesised that the expression of the triticale *Gc* gene suppressor had an influence on the semi-fertility of hybrids but did not inhibit the chromosome rearrangements. This paper also describes the double haploid production, which enabled to obtain plants with two identical copies of triticale chromosomes with translocations of *Aegilops* chromatin segments.

## Introduction

Hexaploid triticale (×*Triticosecale* Wittm. ex A. Camus; 2n = 6x = 42, AABBRR) is a human-made cereal that was supposed to combine the yield potential of wheat (*Triticum aestivum* L.; 2n = 6x = 42, AABBDD) with the resistances and undemanding nature of rye (*Secale cereale* L.; 2n = 2x = 14, RR) in one plant. The breeding efforts aimed to develop a cereal adapted to dry locations, with higher protein content and a feeding quality similar to wheat. However, the synthetic character of this cereal resulted in narrow genetic variability. Moreover, the races of pathogens and pests managed to adapt to this new cereal and begun to break its resistance or tolerance abilities (Kwiatek et al. 2015a, b). To face this problem, there is a need to improve the genetic diversity of triticale by the transfer of desirable genes from wild relatives.

Distant crossing between alien species and wheat or triticale is often disturbed because of crossability barriers, such as different ploidy level of the parental components and the expression of the *Ph1* gene (located on chromosome 5B), responsible for homologues chromosome pairing during meiosis (Riley and Chapman 1958; Griffiths et al. 2006; Lukaszewski and Kopecký 2010). There are several ways that induce the transfer of the chromosomes carrying the gene(s) of interests, including mutagenesis or the exploitation of the mechanisms that allow the homoeologous chromosomes to pair and recombine (absence of *Ph1* gene or the presence of its recessive mutation *ph1b*), which are widely used to obtain the introgressive forms of wheat. The adaptation of the cereal recombinants into elite background is the crucial issue to deal with. Therefore, there is a need to find a natural process that will enable the chromosome segments of wild relatives to be transferred in a targeted manner into a wide range of cultivated species. In *Aegilops* species, a group of chromosomes called gametocidal (*Gc*) chromosomes are known to remain in wheat hybrid plants in a selfish way. The *Gc* chromosomes were first isolated during the production of alien cytoplasm substitution lines or alien chromosome addition lines in common wheat (Endo 1978). The expression of *Gc* factors brings on the sterility of gametes without *Gc* chromosomes, while the gametes with *Gc* chromosomes are fertile (Nasuda et al. 1998). *Gc* factors cause extensive chromosome breakage, which results in the induction of non-functional gametes and exclusive transmission of the *Gc* chromosome to the offspring (Nasuda et al. 1998). Sometimes, the *Gc* factor induces lower levels of chromosome breakage, which can be tolerated considering the polyploid nature of wheat and results in the formation of functional gametes (Nasuda et al. 1998). According to the literature, there are two phenotypes connected with the mechanism responsible for the preferential transmission of the *Gc* chromosomes (Endo 1990; Tsujimoto 2005). The first phenotype induces chromosome breakages, while the second prevents chromosome breakages. When both elements are present, the chromosome aberrations do not occur in gametes, because the gametocidal action is neutralised by the inhibitor. What is more, Friebe et al. (2003) reported the development of a knock-out wheat mutant carrying the *Gc2* locus on *Ae. sharonensis* chromosome 4S^sh^, which has lost the chromosome fragmentation function, but has retained the inhibitor element. The *Gc* system exploitation could be an effective way to induce chromosomal rearrangements and, what is better, it is conceived as safer and easier to handle than mutagens; however, the effectiveness of the *Gc* system in inducing gene mutations is not known (Endo 2007). Nonetheless, the molecular marker mapping enabled to localise *Gc* loci on a region proximal to a block of sub-telomeric heterochromatin on chromosome 4S^sh^L of *Ae. sharonensis* (Knight et al. 2015). *Gc* chromosomes are derived from different genomes (C, S, S^l^ and M^g^) and belong to three different homoeologous chromosome groups 2, 3 and 4 of the *Aegilops* genus (reviewed by Endo 2007).

*Aegilops ovata* Roth. (syn. *Ae. geniculata*; van Slageren 1994) is a tetraploid (2n = 4x = 28, U^g^U^g^M^g^M^g^). The U^g^ genome originated from the U genome of the diploid species *Ae. umbellulata* Zhuk. (2n = 2x = 14, UU) and the M^g^ genome was derived from the M genome of *Ae. comosa* Sm. in Sibth. & Sm. (2n = 2x = 14, MM) (Kihara 1954). *Aegilops ovata* has a wide distribution and is native to the Mediterranean, Middle East and southern parts of Russia and Ukraine. The *Gc* factor was localised on the 4M^g^ chromosome (Kynast et al. 2000). *Aegilops ovata* is a valuable source of resistance genes for biotic and abiotic stresses (Schneider et al. 2008), which can be used for improving cultivated cereals, such as wheat (Friebe et al. 2000), rye (Apolinarska et al. 2010; Kwiatek et al. 2012) and triticale (Kwiatek et al. 2015a, b).

The main aim of this work was to induce the chromosome translocations between *Ae. ovata* (UUMM) and triticale (AABBRR) genomes by the gametocidal system. In this paper, we present an evaluation of the transmission rate of *Aegilops* chromatin in the triticale genomic background. We have used *Ae. ovata* × *S. cereale* amphiploids as parental forms to cross with triticale. The double haploid lines were obtained from fertile or semi-fertile F_2_ hybrids in order to stabilise the *Aegilops* chromatin in the triticale background. Genomic/fluorescence in situ hybridisation (GISH/FISH) techniques were applied to identify the genomes and particular chromosomes.

## Materials and methods

### Plant material

**Fig. 1:**
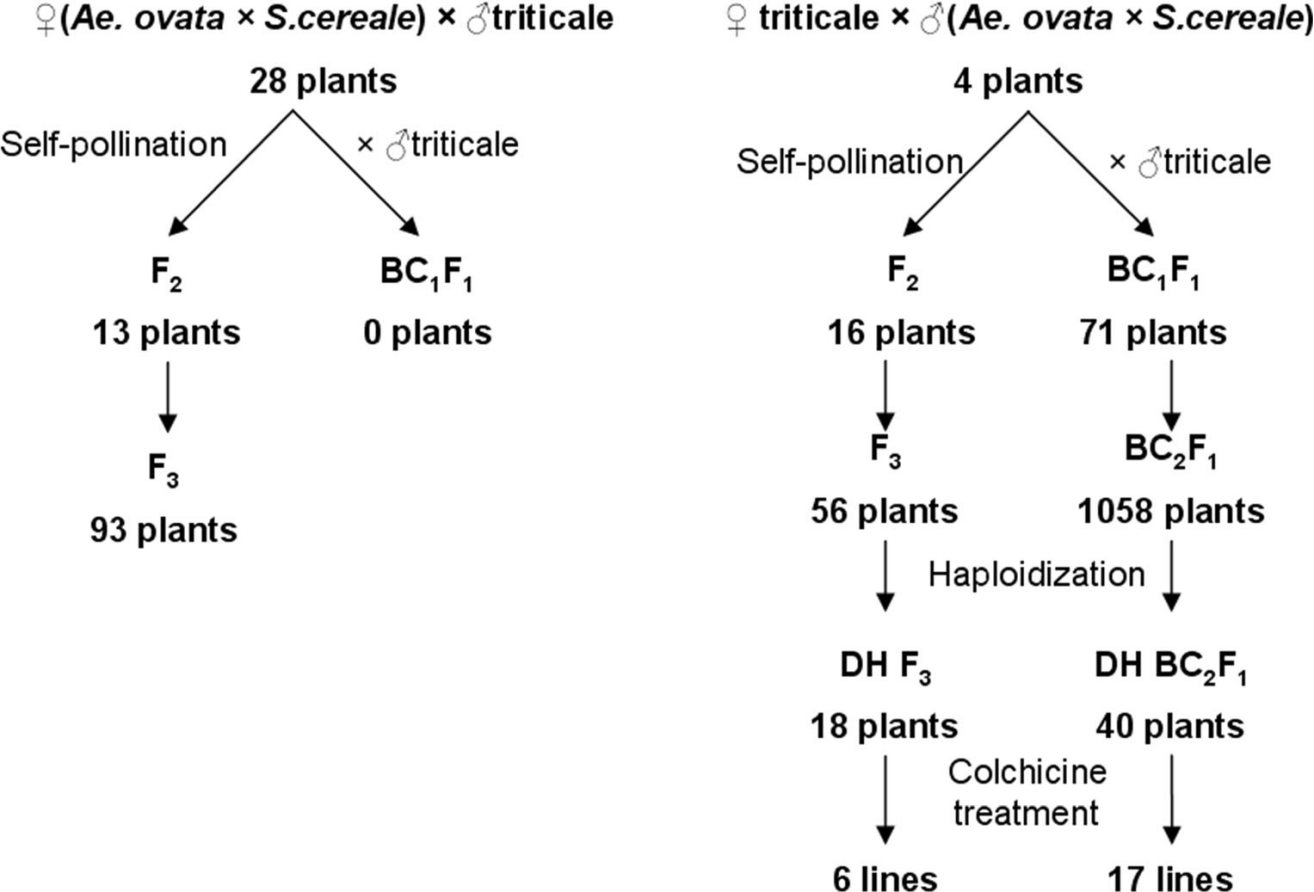
The scheme of subsequent crosses between *Aegilops ovata* × *Secale cereale* amphiploid forms and triticale cv. ‘Moreno’

Glasshouse experiments were carried out at the Institute of Plant Genetics, Polish Academy of Sciences in Poznań, Poland. Seeds of *Ae. ovata* (To36; 2n = 4x = 28; UUMM) were received from the collection of Professor Moshe Feldman (Department of Plant Science, The Weizmann Institute of Science, Rehovot, Israel). The *Ae. ovata* × *S. cereale* amphiploids (UUMMRR, 2n = 6x = 42) were obtained by Wojciechowska and Pudelska (2002). The F_1_ (*Ae. ovata* × *S. cereale*) × triticale hybrids were obtained by reciprocal crossing with triticale cv. ‘Moreno’ received from DANKO Breeding Company (DANKO Hodowla Roślin Sp. z o.o., Choryń, Poland). The following steps and results of offspring production are described in Fig. 1 and Table 1.

**Table 1:** Chromosome composition of *Aegilops ovata* × *Secale cereale* (AoSc) × triticale var. ‘Moreno’ hybrids. ^tM^: chromosomes of triticale carrying M-genome translocations

Generation	Number of plants analysed (total)	Total number of chromosomes	*Ae. ovata* chromosomes		Triticale chromosomes
			U-genome	M-genome	
F_1_ AoSc × triticale	28 (28)	42	7	7	28
F_1_ triticale × AoSc	4 (4)	42	7	7	28
F_2_ AoSc × triticale	13 (13)	38–41	2–4	3–4	18–20
F_2_ triticale × AoSc	16 (16)	41–42	0–3	0–5	19–28
BC_1_F_1_ triticale × AoSc	71 (71)	40–42	0	0	26–28^tM^
F_3_ AoSc × triticale	50 (93)	34–42	0	0	20–28
F_3_ triticale × AoSc	56 (56)	40–42	0	0	26–28^tM^
BC_2_F_1_ triticale × AoSc	50 (1058)	40–42	0	0	26–28^tM^
DH F_3_ triticale × AoSc	12 (6 lines)	42	0	0	28^tM^
DH BC_2_F_1_ triticale × AoSc	34 (17 lines)	42	0	0	28^tM^

### Evaluation of pollen vitality, crossing and crossing efficiency of hybrids

The pollen grains were stained with 2 % acetocarmine in glycerine (vol. 1:1) for the presence of cytoplasm. The evaluation of pollen vitality was made using an Olympus BX60 microscope.

Maternal components were emasculated to avoid self-fertilisation in order to cross with the pollen of triticale cv. ‘Moreno’. The emasculated flowers were counted and pollinated with freshly collected pollen of triticale within a period of 3 months (April–June). The percentage ratio of the total amount of seeds from each plant with the total amount of pollinated flowers of each plant was calculated. The mean of the crossing efficiency of each generation of hybrids was established.

### Double haploid production

The tillers from donor plants were cut at the microspore stage and cold treated at 4 °C for 6–9 days in mineral salt medium N6 (Chu et al. 1975) +2.0 mg/l 2,4-D in the dark. The anthers were cultured on the C17 medium (Wang and Chen 1983) +2 mg/l 2,4-D + 0.5 mg/l KIN + 90 g/l of maltose in darkness at 28 °C. Androgenic structures developed from the microspores were placed in the regeneration medium 190–2 (Zhuang and Xu 1983). Regeneration was induced at 22 °C, in continuous light for 12 h per day. The ploidy level was evaluated on the basis of gDNA content in leaves tissue using flow cytometry. Chromosome doubling was obtained using 0.1 % colchicine solution supplemented with 4 % DMSO and 25 mg/l GA_3_. Afterwards, the plants were placed in the vernalisation chamber for six weeks and then located in the glasshouse until harvest.

### Chromosome preparation

Germination, metaphase accumulation and fixation procedures were carried out according to Kwiatek et al. (2015a). Maceration was made in 0.2 % (*v*/*v*) Onozuka R-10 and Calbiochem cytohelicases (1:1 ratio) and 20 % pectinase (Sigma) in 10 mM citrate buffer (pH 4.6) at 37 °C for 30 min and stopped by washing two times for 10 min each in citrate buffer and in H_2_O. Chromosome preparations were made according to the protocol reported by Heckmann et al. (2014), with modifications made by Hesse (IPK Gatersleben, Germany, personal comm.). Root tips were placed on a slide in a drop of ice-cold 60 % acetic acid and dispersed with a metal needle. Ice-cold acetic acid (60 %) was added to the cell suspension, mixed and kept for 2 min at room temperature. Next, ice-cold 60 % acetic acid was added and the slide was placed on a heating table (Medax) for 2 min at 48 °C. The slide was removed from the hot plate and 200 μl ice-cold ethanol–acetic acid (3:1, *v*/*v*) was added to briefly wash the slides. The slide was placed in 60 % acetic acid for 10 min, followed by briefly washing in 96 % ethanol and then air dried. Hybrids were grown in the nursery and their meiotic behaviour was analysed in pollen mother cells (PMCs) at metaphase I (MI) of meiosis. Anthers of the hybrids containing PMCs at MI were fixed in 1:3 (*v*/*v*) acetic acid/ethanol and stored at −20 °C for a maximum of 2 months. MI of meiosis preparations were made according to Zwierzykowski et al. (2008). The anthers were squashed in 45 % acetic acid and the slides were stored at 4 °C until in situ hybridisation.

### Probe labelling

Total genomic DNA was extracted from fresh leaves of *Ae. umbellulata* (UU), *Ae. comosa* (MM) and triticale ‘Moreno’ (AABBRR) using GeneMATRIX Plant & Fungi DNA Purification Kit (EURx Ltd.). Genomic DNA from *Ae. umbellulata* and *Ae. comosa* were labelled by nick translation (using the Nick Translation Kit, Roche, Mannheim, Germany) with digoxigenin-11-dUTP (Roche) or tetramethyl-5-dUTP-rhodamine (Roche), respectively. Blocking DNA from triticale was sheared by boiling for 30–45 min and used at a ratio of 1:50 (probe:block). The 5S rDNA probe was amplified from the wheat clone pTa794 (Gerlach and Dyer 1980) by polymerase chain reaction (PCR) with tetramethyl-rhodamine-5-dUTP (Roche) using universal M13 ‘forward’ (5′-CAG GGT TTT CCC AGT CAC GA-3′) and ‘reverse’ (5′-CGG ATA ACA ATT TCA CAC AGG A-3′) sequencing primers. The PCR reactions were performed as follows: 94 °C for 1 min, 39 cycles of 94 °C for 40 s, 55 °C for 40 s and 72 °C for 90 s, and 72 °C for 5 min. The 25S rDNA probe was made by nick translation of a 2.3-kb *Cla*I sub-clone of the 25-5.8-18S rDNA coding region of *Arabidopsis thaliana* (Unfried and Gruendler 1990) with digoxigenin-11-dUTP (Roche). It was used for the detection of 25-5.8-18S rDNA loci. The pSc119.2 repetitive DNA sequence, supplied by Dr. Kubalaková (Laboratory of Molecular Cytogenetics and Cytometry, Institute of Experimental Botany, Olomouc, Czech Republic), was amplified and labelled by PCR with digoxigenin-11-dUTP (Roche) using universal M13 primers (Vrána et al. 2000). The pAs1 probe was amplified by PCR from the genomic DNA of *Ae. tauschii* and labelled with digoxigenin-11-dUTP (Roche), according to the protocol reported by Nagaki et al. (1995).

### In situ hybridisation

GISH was carried out according to Kwiatek et al. (2012), with modifications. Multi-colour GISH was carried out using the U-genome probe (from *Ae. umbellulata*), M-genome probe (from *Ae. ovata*) and unlabelled triticale genomic DNA, which was used as a specific blocker. The GISH mixture (40 μl per slide), containing 50 % formamide, 2 × SSC, 10 % dextran sulphate, 90 ng each of the genome probes and 4.5 μg blocking DNA, was denatured at 75 °C for 10 min and stored on ice for 10 min. Chromosomal DNA was denatured in the presence of the hybridisation mixture at 75 °C for 5 min and allowed to hybridise overnight at 37 °C. For detection of the hybridisation signals, anti-digoxigenin IgG conjugated with FITC (Roche) was used. After documentation of the FISH sites, the slides were washed according to Heslop-Harrison (2000) (2 × 45 min in 4 × SSC Tween, 2 × 5 min in 2 × SSC, at room temperature). To recognise the particular chromosomes, we used four probes for in situ hybridisation on the same chromosome preparations in two subsequent FISH runs. The first FISH was carried out according to Książczyk et al. (2011), with minor modifications of Kwiatek et al. (2013), using 25S (used for the detection of 25-5.8-18S rDNA loci) and 5S rDNA (pTa794). The hybridisation mixture (40 μl per slide) contained 90 ng of each probe in the presence of salmon sperm DNA, 50 % formamide, 2 × SSC and 10 % dextran sulphate, and was denatured at 75 °C for 10 min and stored on ice for 10 min. The second FISH, with pSc119.2 and pAs1 (labelled with digoxigenin-11-dUTP and tetramethyl-rhodamine-5- dUTP, respectively), was performed with the same conditions after reprobing. The chromosomal DNA denaturation, hybridisation and immunodetection conditions were the same as those mentioned above. Digoxigenin detection was carried out using anti-digoxigenin-fluorescein antibody (Roche). FISH was carried out to study the mitotic chromosomes of root meristems. On the other hand, GISH was used to examine both the mitotic chromosomes of root meristems and meiotic chromosomes of PMCs. Mitotic and meiotic (MI) cells were examined with an Olympus XM10 CCD camera attached to an Olympus BX 61 automatic epifluorescence microscope. Image processing was carried out using Olympus Cell-F (version 3.1; Olympus Soft Imaging Solutions GmbH, Münster, Germany) imaging software and PaintShop Pro X5 software (version 15.0.0.183; Corel Corporation, Ottawa, Canada). The identification of particular chromosomes was made by comparing the signal pattern of 5S rDNA, 25S rDNA, pSc119.2 and pAs1 probes according to a previous study (Kwiatek et al. 2013) and similar cytogenetic analysis (Cuadrado and Jouve 1994; Schneider et al. 2003, 2005; Wiśniewska et al. 2013).

## Results

### Evaluation of pollen viability and crossing efficiency

The mean percentage of pollen viability of *Ae. ovata* × *S. cereale* amphiploids was 64.4 %, while the pollen viability of triticale var. ‘Moreno’ ranged from 86 to 94 %. Reciprocal hybridisation was done to obtain hybrid forms between triticale ‘Moreno’ (AABBRR) and *Ae. ovata* × *S. cereale* (UUMMRR) amphiploids. Triticale pollen was used to pollinate 168 flowers of *Ae. ovata* × *S. cereale* plants. Twenty-eight obtained seeds were germinated, self-pollinated and back-crossed with triticale. The mean pollen viability of (*Ae. ovata* × *S. cereale*) × triticale F_1_ hybrids was 4.82 %. 13 F_2_ and 0 BC_1_F_1_ seeds were harvested after self-pollinating or back-crossing of 212 and 468 flowers, respectively. The pollen viability of F_2_ plants was 32.42 %. Self-pollination of 334 flowers enabled to obtain 93 seeds of the F_3_ generation. Simultaneously, 354 flowers of triticale ‘Moreno’ were pollinated by the pollen of *Ae. ovata* × *S. cereale* forms. Four F_1_ seeds were germinated and evaluated using FISH/GISH analysis. The F_1_ plants produced pollen grains with 9.09 % viability. Self-pollination of 248 flowers resulted in 16 seeds of F_2_ progeny. Back-crossing of 334 flowers of F_1_ hybrids with the triticale ‘Moreno’ pollen gave 71 seeds of the BC_1_F_1_ hybrid generation. The mean pollen viability was 24.20 % for F_2_ hybrids and 50.93 % for BC_1_F_1_ plants. Afterwards, the F_2_ hybrids (381 flowers) were self-pollinated and BC_1_F_1_ plants (1518 flowers) were pollinated with triticale pollen. 56 F_3_ and 1058 BC_2_F_1_ seeds were harvested, respectively.

### Anther cultures in *Aegilops* triticale hybrids

Overall, 2896 anthers from F_3_ ‘Moreno’ × (*Ae. ovata* × *S. cereale*) plants and 3204 anthers from BC_2_F_1_ hybrids were used to produce 3461 (119.5 per 100 anthers) and 4444 (138.7 per 100 anthers) androgenic structures, from which 18 (0.62) and 40 (1.3 per 100 anthers) haploid plants were obtained, respectively. Thereafter, 6 and 17 double haploid lines (respectively) of introgressive triticale were generated (Fig. 1) using the colchicine solution to double the ploidy level.

### Transmission of *Aegilops ovata* chromatin in triticale hybrids

**Fig. 2:**
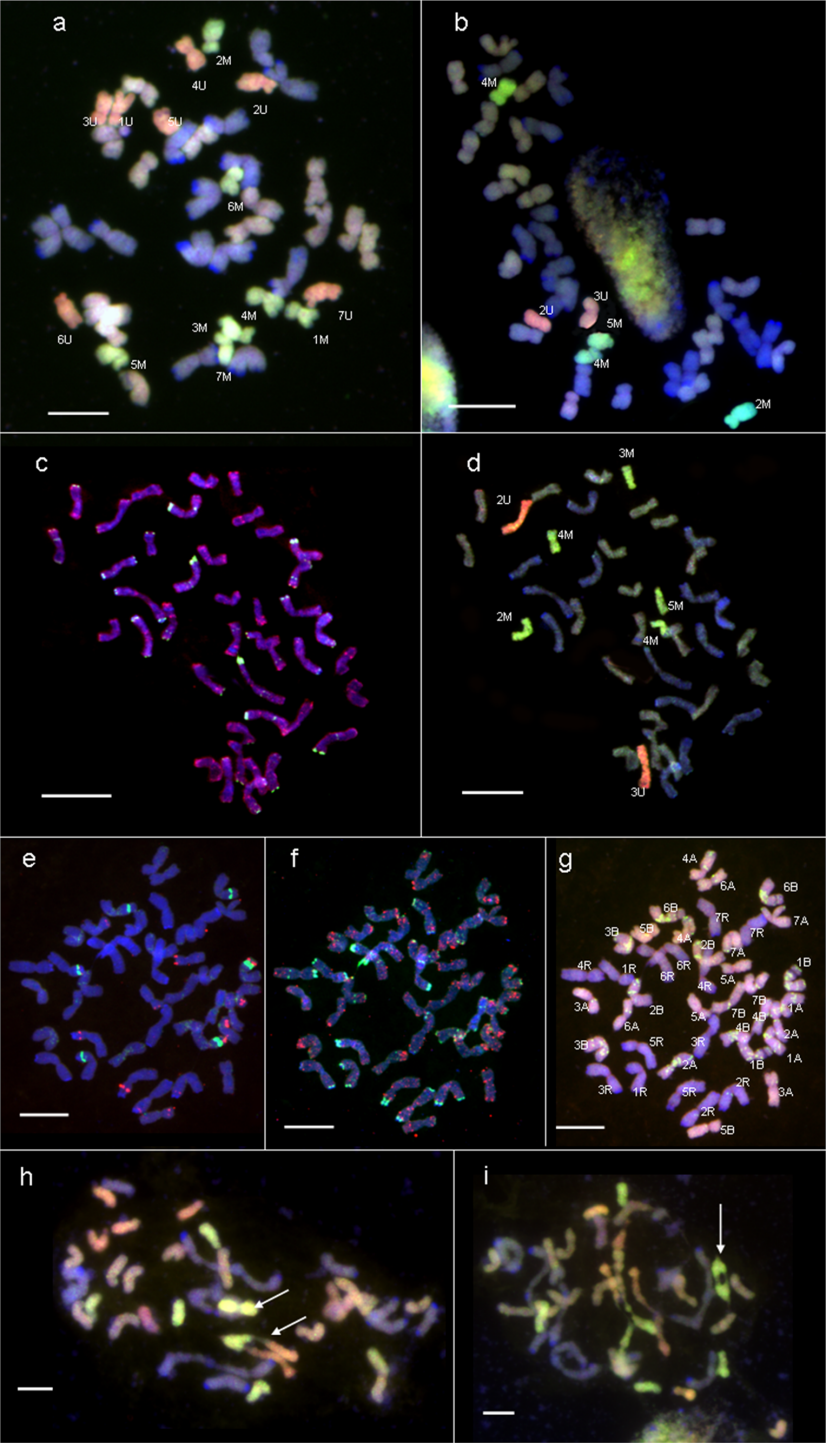
**a** Genomic in situ hybridisation (GISH) experiment showing seven U-genome (*red*) and seven M-genome (*green*) chromosomes of *Aegilops ovata* and 28 triticale chromosomes (*grey* and *blue*) of F_1_ plants during metaphase I (MI) of mitosis. **b** GISH experiment showing mitotic chromosomes of U-genome (*red*), M-genome (*green*) and triticale genomes (*grey* and *blue*) in F_2_ triticale × (*Ae. ovata* × *Secale cereale*) plants. **c** Fluorescence in situ hybridisation (FISH) experiment presenting the location of pSc119.2 (*green*) and pAs1 (*red*) probes in mitotic chromosomes of F_2_ (*Ae. ovata* × *S. cereale*) × triticale plants. **d** GISH experiment showing chromosomes of U-genome (*red*), M-genome (*green*) and triticale genomes (*grey* and *blue*) during mitosis of the same F_2_ (*Ae. ovata* × *S. cereale*) × triticale plants. Mitotic chromosomes of BC_1_F_1_ triticale × (*Ae. ovata* × *S. cereale*) plants analysed by: **e** FISH with 5S and 35S rDNA probes, **f** FISH with pSc119.2 (*green*) and pAs1 (*red*) probes and **g** GISH with U-genome (*red*), M-genome (*green*) and triticale (*grey* and *blue*) genomic probes. **h** F_1_ plant during MI of meiosis carrying M-genome bivalent and UMU-genomes trivalent (*arrows*) and **i** F_2_ (*Ae. ovata* × *S. cereale*) × triticale plant carrying 4M chromosome bivalent (*arrow*). Scale bar: 10 μm

The chromosome constitution of both F_1_ (triticale as a male or female parent) combinations was the same and consist of: 14 chromosomes of *Ae. ovata* (seven chromosomes of U-genome and seven chromosomes of M-genome) and 28 chromosomes of triticale (14 chromosomes of R-genome and 14 chromosomes of A- and B-genomes; Table 1; Fig. 2a). The total number of chromosomes was 42. In 13 F_2_ plants of the (*Ae. ovata* × *S. cereale*) × triticale combination, the number of chromosomes ranged from 38 to 41. The range of both U- and M-genome chromosomes was 2–4. In each of the 13 plants, a pair of 4M chromosomes was identified (Table 2; Fig. 2c, d). Chromosomes 1U, 5U, 7U, 1M, 6M and 7M were eliminated.

**Table 2:** Frequencies of *Aegilops ovata* chromosomes in F_2_ hybrid plants

Plant (number of chromosomes)	1U	2U	3U	4U	5U	6U	7U	Σ_U_	1M	2M	3M	4M	5M	6M	7M	Σ_M_
F_2_ (*Ae. ovata* × *S. cereale*) × triticale																
1 (38)	0	1	1	1	0	0	0	3	0	1	0	2	1	0	0	4
2 (38)	0	1	1	1	0	1	0	4	0	1	1	2	0	0	0	4
3 (38)	0	1	1	1	0	0	0	3	0	1	1	2	0	0	0	4
4 (38)	0	1	1	0	0	1	0	3	0	1	0	2	0	0	0	3
5 (39)	0	1	0	1	0	0	0	2	0	1	1	2	0	0	0	4
6 (40)	0	1	0	1	0	1	0	3	0	1	0	2	0	0	0	3
7 (40)	0	1	0	1	0	1	0	3	0	1	0	2	0	0	0	3
8 (40)	0	1	0	1	0	0	0	2	0	1	0	2	0	0	0	3
9 (41)	0	1	0	1	0	0	0	2	0	1	0	2	1	0	0	4
10 (41)	0	1	1	1	0	0	0	3	0	1	0	2	0	0	0	3
11 (41)	0	1	1	0	0	0	0	2	0	1	0	2	1	0	0	4
12 (41)	0	1	1	1	0	0	0	3	0	1	0	2	1	0	0	4
13 (41)	0	1	0	1	0	0	0	2	0	1	0	2	0	0	0	3
Σ	0	13	7	11	0	4	0		0	13	3	26	4	0	0	
Frequency	0	1	0.5	0.8	0	0.3	0		0	1	0.2	2	0.3	0	0	
F_2_ triticale × (*Ae. ovata* × *S. cereale*)																
1 (41)	0	1	0	1	0	0	0	2	0	1		1	0	0	0	2
2 (41)	0	1	1	1	0	0	0	3	0	1	1	1	0	0	0	3
3 (41)	0	1	1	1	0	0	0	3	0	1	1	1	0	0	0	3
4 (41)	0	0	0	0	0	0	0	0	0	0	0	0	0	0	0	0
5 (41)	0	1	1	1	0	0	0	3	0	1	1	1	0	0	0	3
6 (41)	0	1	1	0	0	0	0	2	0	1	1	2	0	0	0	4
7 (42)	0	1	1	1	0	0	0	3	0	1	1	1	0	0	0	3
8 (42)	0	1	1	0	0	0	0	2	0	1	1	2	1	0	0	5
9 (42)	0	0	0	0	0	0	0	0	0	0	0	0	0	0	0	0
10 (42)	0	0	1	1	0	0	0	2	0	1	1	1	0	0	0	3
11 (42)	0	0	0	0	0	0	0	0	0	0	0	0	0	0	0	0
12 (42)	0	1	1	1	0	0	0	3	0	0	0	0	0	0	0	0
13 (42)	0	1	1	1	0	0	0	3	0	1	0	1	0	0	0	2
14 (42)	0	1	1	1	0	0	0	3	0	1	1	2	0	0	0	4
15 (42)	0	1	1	1	0	0	0	3	0	1	1	1	0	0	0	3
16 (42)	0	0	0	0	0	0	0	0	0	1	1	1	0	0	0	3
Σ	0	11	11	10	0	0	0		0	12	10	15	1	0	0	
Frequency	0	0.7	0.7	0.6	0	0	0		0	0.8	0.6	0.9	0.1	0	0	

On the other hand, the chromosome constitution of F_2_ triticale × (*Aegilops ovata* × *S. cereale*) hybrids was different. The total number of chromosomes ranged between 41 and 42. The lack of *Aegilops* chromosomes was revealed in three plants. The range of *Aegilops* chromosomes in the remaining 15 plants was 0–3 and 0–5 for U- and M-genomes, respectively (Fig. 2b). The most common were 4M and 2M chromosomes. In three plants, a pair of 4M chromosomes was identified. *Aegilops* chromosomes from the 1st, 6th and 7th homoeologous groups were not identified (Table 2).

Complete *Aegilops* chromosomes were absent in each of the 71 BC_1_F_1_ plants of the triticale × (*Ae. ovata* × *S. cereale*) combination; however, each of the A- and B-genome chromosomes of triticale carried small segments or distant signals on M-genome chromatin, except for the 5A chromosome pair. Clear M-genome probe signals were also detected on chromosomes of the 2R pair (Fig. 2e, f, g). The total number of chromosomes in BC_1_F_1_ plants was 40 (in 23 plants), 41 (12 plants) and 42 (36 plants). In the nullisomic or monosomic plants of triticale hybrids, the 2A, 2B or 3B chromosome pairs or singe chromosomes were missing.

The chromatin of *Ae. ovata* was completely eliminated in 50 F_3_ plants of the (*Ae. ovata* × *S. cereale*) × triticale combination. Those plants carried different numbers of triticale chromosomes, ranging between 34 and 42. The 2B, 3B, 4B and 6B chromosomes or chromosome pairs were lacking in these forms. Complete *Ae. ovata* chromosomes were also absent in 56 F_3_ and 50 BC_2_F_1_ plants of the triticale × (*Aegilops ovata* × *S. cereale*) combination. However, the chromosomes of A- and B-genomes carried M-genome chromatin translocations, which were similar to those observed in BC_1_F_1_ plants.

**Fig. 3:**
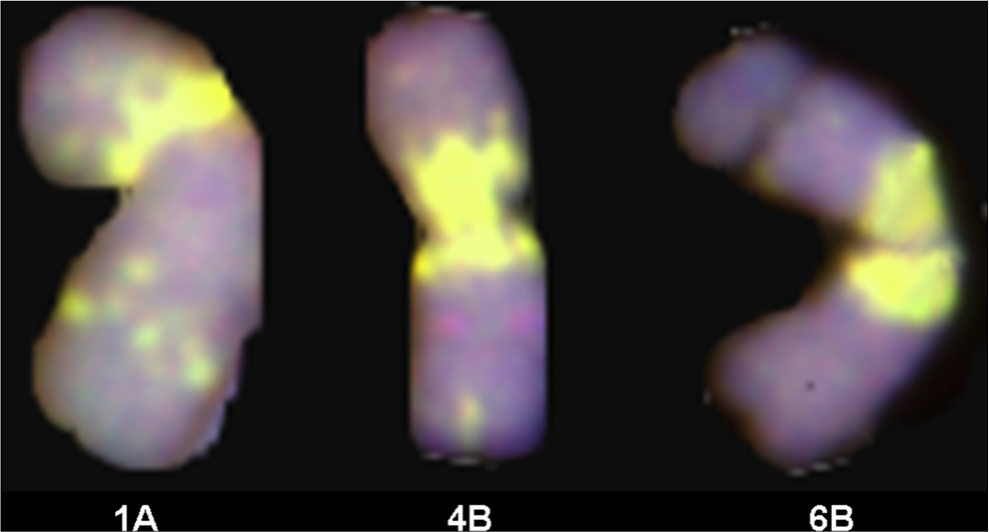
GISH showing the location of M-genome chromosome segments of *Ae. ovata* (*green*) on the 1A, 4B and 6B somatic chromosomes of a DH BC_1_F_2_ triticale × (*Ae. ovata* × *S. cereale*) plant

**Fig. 4:**
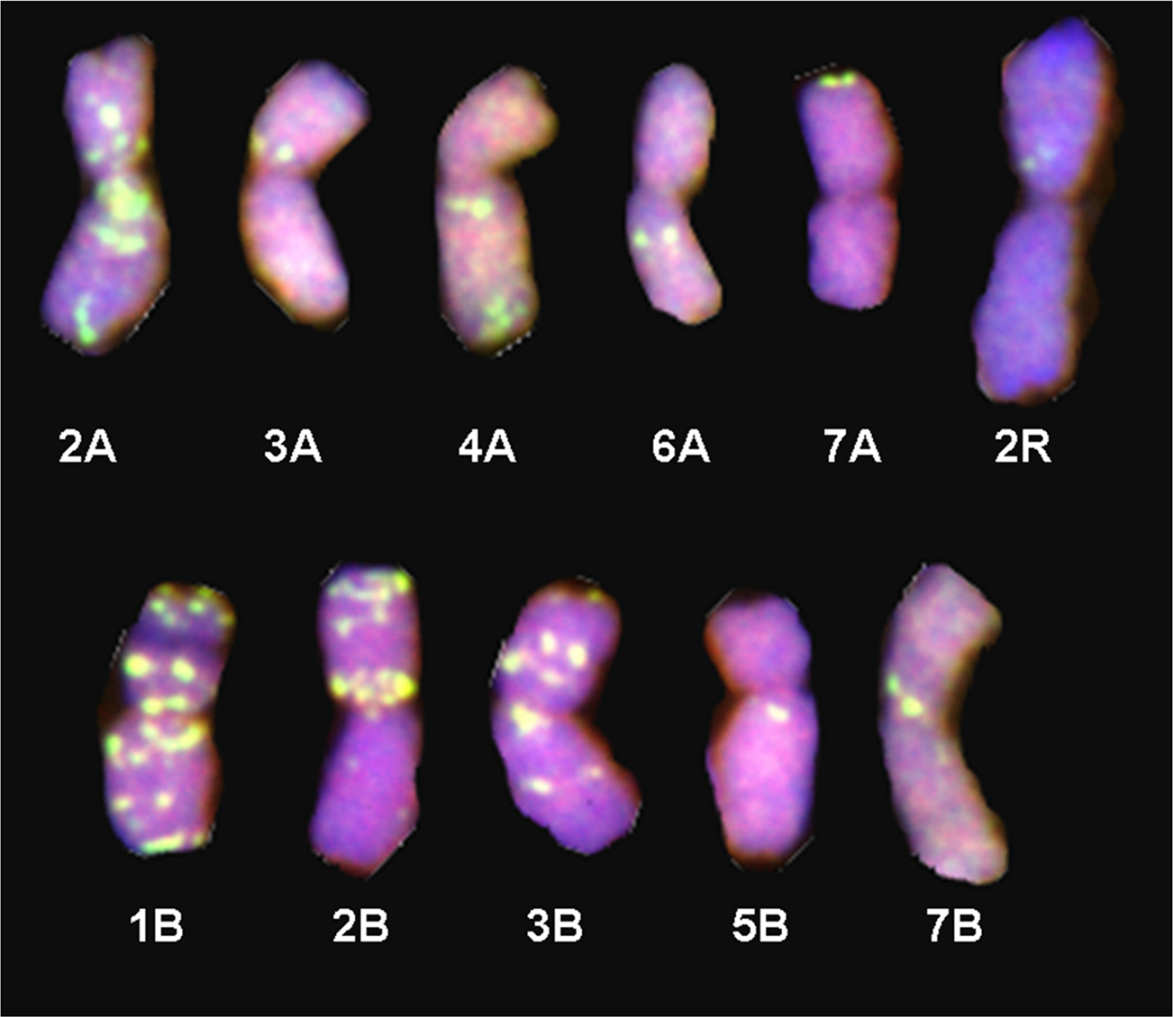
GISH showing the location of M-genome repetitive sequences of *Ae. ovata* (*green*) on the individual somatic chromosomes of a DH BC_1_F_2_ triticale × (*Ae. ovata* × *S. cereale*) plant

Finally, the 12 and 34 DH plants obtained from F_3_ and BC_2_F_1_ hybrids of the triticale × (*Aegilops ovata* × *S. cereale*) combination carried 42 chromosomes. The most abundant signals of the M-genome probe appeared on 1A, 2A, 1B, 3B, 4B, 6B and in the short arm of 2B. The M-genome chromosome segments were located in pericentromeric regions on the 2A, 4B and 6B chromosomes of triticale (Fig. 3). Clear but single signals were observed on the 3A, 4A, 6A, 7A, 5B, 7B and 2R chromosomes as well (Fig. 4). Moreover, M-genome landmarks were dispersed on the 1A, 1B and 3B chromosomes. Furthermore, M-genome signals were also observed in telomeric regions of the 7AS, 1BS, 1BL, 2AL, 3BL, 4BL and 6BL chromosome arms.

### *Aegilops ovata* chromosome behaviour at metaphase I of meiosis of F_1_, F_2_ and BC_1_F_1_ hybrids

The meiotic analyses were done in two initial generations of hybrids, where *Ae. ovata* chromosomes were established. The chromosome configurations of 32 F_1_ plants during MI of meiosis were in parallel with the theoretical assumptions. Seven univalents of the U-genome as well as seven univalents of the M-genome were observed. The number of A- and B-genome chromosomes was haploid as well. Furthermore, the R-genome chromosomes paired and created rod or ring bivalents. However, in two other F_1_ plants from the (*Ae. ovata* × *S. cereale*) × triticale combination, one ring bivalent of the M-genome was detected. Moreover, those hybrids also carried a trivalent that consisted of two U-genome chromosomes linked with one M-genome chromosome (Table 3; Fig. 2h). In the F_2_ generation of the (*Ae. ovata* × *S. cereale*) × triticale combination, the number of *Ae. ovata* univalents ranged between 0 and 8. One ring bivalent of the M-genome was detected (Fig. 2i). Moreover, the diverse types of intergenomic bivalents, trivalents and tetravalents were observed. The frequencies are shown in Table 3. On the other hand, the *Ae. ovata* univalents were more abundant in the F_2_ generation of the triticale × (*Ae. ovata* × *S. cereale*) combination, while the frequencies of bivalents and multivalents were lower (Table 3). What is more, the M-genome bivalents were not observed.

**Table 3:** Frequency of *Ae. ovata* (UM) chromosome configurations in metaphase I (MI) of meiosis of triticale (T) hybrids

Plant number (number of chromosomes)	Number of PMCs	Mean (range) of chromosome configurations at metaphase I										
		Univalents		Bivalents					Trivalents			Tetravalents
		U	M	U/U	M/M	U/M	U/T	M/T	UMU	UTT	MTT	U/T/T/M
F_1_ (*Ae. ovata* × *S. cereale*) × triticale												
28 (42)	280	7 (7)	6.86 (4–7)	0 (0)	0.07 (0–1)	0 (0)	0 (0)	0 (0)	0.07 (0–1)	0 (0)	0 (0)	0 (0)
F_1_ triticale × (*Ae. ovata* × *S. cereale*)												
4 (42)	40	7 (7)	7 (7)	0 (0)	0 (0)	0 (0)	0 (0)	0 (0)	0 (0)	0 (0)	0 (0)	0 (0)
F_2_ (*Ae. ovata* × *S. cereale*) × triticale												
4 (38)	40	1.83 (0–4)	1.85 (1–4)	0	1 (1)	0.75 (0–1)	0.25 (0–1)	0.15 (0–1)	0 (0)	0.63 (0–1)	1.25 (0–2)	0.25 (0–1)
1 (39)	10	1.60 (0–4)	1.90 (1–4)	0	1 (1)	0.70 (0–1)	0.30 (0–1)	0.20 (0–1)	0 (0)	0.60 (0–1)	1.20 (0–2)	0.20 (0–1)
3 (40)	30	1.77 (0–4)	1.86 (1–4)	0	1 (1)	0.72 (0–1)	0.30 (0–1)	0.27 (0–1)	0 (0)	0.67 (0–1)	1.27 (0–2)	0.27 (0–1)
5 (41)	50	1.72 (0–4)	1.68 (1–4)	0	1 (1)	0.66 (0–1)	0.28 (0–1)	0.18 (0–1)	0 (0)	0.68 (0–1)	1.26 (0–2)	0.26 (0–1)
F_2_ triticale × (*Ae. ovata* × *S. cereale*)												
6 (41)	60	1.83 (0–3)	2.67 (0–4)	0 (0)	0 (0)	0.33 (0–1)	0 (0)	1.33 (0–2)	0 (0)	0 (0)	0.63 (0–1)	0 (0)
10 (42)	100	1.93 (0–3)	2.29 (0–4)	0 (0)	0 (0)	0 (0)	0 (0)	0.76 (0–1)	0 (0)	0 (0)	0.68 (0–1)	0 (0)

## Discussion

Considering the narrow genetic diversity of triticale, it is necessary to utilise the wild Triticeae relatives to enrich the genetic pool of this species. The most common method used to transfer genes of agronomic value from wild relatives into cultivated cereals are interspecific hybridisations. Initially, one of the main breeding strategies aiming at triticale improvement was to introduce D-genome chromosomes into 6x triticale. A number of efforts in the production of triticale substitution lines were conducted. The simplest way to obtain the D(A) or/and D(B) substitution lines is an application of octoploid (AABBDDRR) × tetraploid (AARR or BBRR) triticale crosses (Krowlow 1970; Lukaszewski et al. 1984; Apolinarska 1993). Moreover, as well as in wheat breeding, several attempts were made to cross triticale with *Aegilops* species (Gruszecka et al. 1996; Gradzielewska et al. 2012; Kwiatek et al. 2015a, b), which are related to *Triticum* species and represent a large source of useful traits for crop improvement (Schneider et al. 2008). The alien species *Ae. speltoides*, *Ae. cylindrica*, *Ae. sharonensis*, *Ae. longissima* and *Ae. ovata* are reported to posses gametocidal genes, which give rise to chromosomal rearrangements (Endo 2007).

 Up to now, the process of preferential transmission of chromosomes carrying gametocidal (*Gc*) factors that originated from *Aegilops* species were reported only considering the wheat genetic background (Finch et al. 1984; Friebe et al. 1999; Kynast et al. 2000; Endo 2007; Ishihara et al. 2014). Here, we report novel data about the behaviour of *Ae. ovata* chromosomes in the triticale genetic background. It is worth mentioning that *Aegilops* chromosomes were transferred to triticale from a stable and fertile (64.4 %) *Ae. ovata* × *S. cereale* amphiploid form, where *Ae. ovata* was a female parent (Wojciechowska and Pudelska 2002). Therefore, the cytoplasm of the amphiploid originated from *Aegilops* species. Hence, the reciprocal crosses between *Aegilops*–*Secale* forms and triticale were made to evaluate the impact of the cytoplasm on the transmission of the *Ae. ovata* chromatin to hybrid progeny.

 All of the F_1_ plants obtained from both combinations carried the expected haploid number of *Aegilops* chromosomes. These hybrids underwent meiosis, showing, in most cases, 14 univalents of *Ae. ovata*, 14 univalents from A- and B-genomes, and 14 bivalents from the R-genome. However, pairing of M-genome chromosomes was observed in the pollen mother cells of two plants. Additionally, another M-genome chromosome was involved in trivalent formation with two U-genome chromosomes. This phenomena seems to be cryptic, considering that each of the *Aegilops* chromosomes appeared in one copy during the MI of F_1_ hybrids. It could be possible that homoeologous pairing took place to balance the lack of homologues. The F_2_ and BC_1_F_1_ hybrids were obtained, regardless of chromosome configuration during the MI of meiosis, which caused low pollen fertility as well as decreased crossing ability of F_1_ hybrids.

 All of the F_2_ plants with *Aegilops* cytoplasm carried a pair of 4M chromosomes, which suggested that the preferential transmission of that particular chromosome appeared during both androgenesis and gynogenesis. What is more, those chromosomes paired during the MI of meiosis of each F_2_ plant PMCs (Table 3; Fig. 2i). In contrast, the process of preferential transmission was weaker considering the F_2_ progeny carrying triticale cytoplasm. In this case, only three plants carried a pair of 4M chromosomes. The rest of the F_2_ plants possess one copy of the 4M chromosome. Furthermore, three other plants did not carry any of the *Aegilops* chromosomes (Table 2). Both combinations gave semi-fertile pollen grains, but the pollen viability of F_2_ progeny carrying *Aegilops* cytoplasm (32.42 %) was higher than in the F_2_ hybrids of triticale × (*Ae. ovata* × *S. cereale*).

 On the other hand, back-crossing with the triticale pollen resulted in a lack of BC_1_F_1_ hybrids with *Aegilops* cytoplasm. In contrast, the same crossing performed on F_1_ hybrids with triticale cytoplasm effected many translocations of *Aegilops* chromatin in triticale chromosomes. It could be supposed that the origin of the cytoplasm has an influence on *Aegilops* chromosome transmission. This result also enable to assume the existence of a cytoplasmic factor that increases the effect of 4M chromosome transmission on one hand and causes abortion of a gamete lacking the 4M chromosome on the other. On the contrary, the existence of *Gc* suppressor, which decreases the effect of preferential transmission and makes the gametes lacking the 4M chromosome fertile or semi-fertile, could be considered as a counter-hypothesis. This point of view is in parallel to the observations made by Tsujimoto and Tsunewaki (1985) considering the different level of PMCs viability of monosomic wheat: ‘Chinese Spring’, ‘John Fife’ and ‘Norin26’ carrying additional an *Gc* chromosome 3C of *Ae. triuncialis*. The first two forms showed both male and female semi-sterility (Endo 1978). However, semi-sterility did not appear in the cultivar ‘Norin26’. Moreover, chromosome 3C was preferentially transmitted to the next generation from both paternal and maternal sides in ‘John Fife’, but only from the female side in ‘Chinese Spring’. In the ‘Norin26’ cultivar, the chromosome 3C was transmitted normally as a usual alien monosome without preferential transmission (Endo 1978). Tsujimoto and Tsunewaki (1985) crossed the ‘Chinese Spring’ addition line carrying two 3C chromosomes with the F_1_ progeny of ‘Chinese Spring’ and ‘Norin26’ and obtained the monosomic addition lines, fertile and semi-sterile plants segregated 1:1. On this basis, they suspected that a dominant suppressor gene (*Igc1*) controls the suppression of *Gc* gene expression on chromosome 3C. In our study, the genetic background of cultivar ‘Moreno’ provided the semi-sterility of PMCs but did not prevent the fragmentation of *Aegilops* chromosomes in F_3_, BC_1_F_1_ and BC_2_F_1_ plants. On this basis, it can be assumed that the suppressing factor showed incomplete expression.

 Analogous conclusions were formed by Tsujimoto and Tsunewaki (1985), who reported that, in the ‘John Fife’ genetic background, both male and female gametes without chromosome 3C were abortive, while in the ‘Chinese Spring’ genetic background, pollen without the *Gc* chromosome functioned and transmitted to the progeny, indicating the expression of an incomplete suppressor in the ‘Chinese Spring’ cytoplasm. The M-genome translocations on chromosomes of F_3_ triticale were located in the pericentromeric region and located on chromosomes 1A, 4B and 6B. The rest of the signals, which were observed on A-, B- and R-genome chromosomes, probably show the distribution of repetitive sequences that are specific for Triticeae species. The existence of classes of widespread repetitive sequences have been reported in the tribe Triticeae (Vershinin et al. 1994). The occurrence of those signals, detected in distant hybrids, indicates the high level of resemblance between the repetitive sequences in selected species. More detailed analysis should be made to establish the precise locations of M-genome chromatin in triticale chromosomes.

 The patterns of M-genome signals were similar in double haploid lines obtained from F_3_ and BC_2_F_1_. The effectiveness of in vitro cultures, treated as the number of plants compared to the number of anthers, is a crucial factor for producing prebreeding germplasm. The number of green haploid plants was 18 for F_3_ (0.62 per 100 anthers) and 40 for BC_2_F_1_ (1.3 per 100 anthers), and was low when compared to similar experiments carried out on triticale, which resulted from 0.4 to even 7.9 green plants per 100 anthers (Ślusarkiewicz-Jarzina and Ponitka 1997, 2003; Ponitka et al. 1999). Double haploids are being produced from triticale in support of breeding programmes. Haploid lines and haploidy on intergeneric hybrids have been successively applied to bridge the D- and R-genomes in the 6x triticale and for creating alien addition lines of F_1_ hybrids (Wang and Hu 1993). In this study, we obtained six DH F_3_ and 17 DH BC_1_F_1_ lines of triticale with maintained translocations of the M-genome from *Ae. ovata*. Considering the homogeneity of DH plants, we transferred the *Aegilops* chromatin into triticale chromosomes using gametocidal gene expression, which induced chromosome rearrangements. Furthermore, we used in vitro anther cultures to generate haploids, which were developed into double haploid plants with two identical copies of triticale chromosomes with *Aegilops* chromatin segments (Fig. 3). The presented modus operandi showed that the *Gc* mechanism can be exploited in the analysis of the biology, structure and transmission of cereal chromosomes. Moreover, the *Gc* chromosomes can also be used in breeding as vectors for the preferential transmission of desirable traits or as inducers for chromosome aberrations.
